# Validity of a multiphase CT-based radiomics model in predicting the Leibovich risk groups for localized clear cell renal cell carcinoma: an exploratory study

**DOI:** 10.1186/s13244-023-01526-2

**Published:** 2023-10-10

**Authors:** Huayun Liu, Zongjie Wei, Yingjie Xv, Hao Tan, Fangtong Liao, Fajin Lv, Qing Jiang, Tao Chen, Mingzhao Xiao

**Affiliations:** 1 Department of Urology, The First Affiliated Hospital of Chongqing Medical University, No. 1 Youyi Road, Yuzhong District, Chongqing, 400016 China; 2 Department of Radiology, The First Affiliated Hospital of Chongqing Medical University, Chongqing, China; 3 Department of Urology, The Second Affiliated Hospital of Chongqing Medical University, Chongqing, China; 4 Department of Pathology, The First Affiliated Hospital of Chongqing Medical University, Chongqing, China

**Keywords:** Radiomics, Clear cell renal cell carcinoma, Leibovich, Tomograph, Prognosis

## Abstract

**Objective:**

To develop and validate a multiphase CT-based radiomics model for preoperative risk stratification of patients with localized clear cell renal cell carcinoma (ccRCC).

**Methods:**

A total of 425 patients with localized ccRCC were enrolled and divided into training, validation, and external testing cohorts. Radiomics features were extracted from three-phase CT images (unenhanced, arterial, and venous), and radiomics signatures were constructed by the least absolute shrinkage and selection operator (LASSO) regression algorithm. The radiomics score (Rad-score) for each patient was calculated. The radiomics model was established and visualized as a nomogram by incorporating significant clinical factors and Rad-score. The predictive performance of the radiomics model was evaluated by the receiver operating characteristic curve, calibration curve, and decision curve analysis (DCA).

**Results:**

The AUC of the triphasic radiomics signature reached 0.862 (95% CI: 0.809–0.914), 0.853 (95% CI: 0.785–0.921), and 0.837 (95% CI: 0.714–0.959) in three cohorts, respectively, which were higher than arterial, venous, and unenhanced radiomics signatures. Multivariate logistic regression analysis showed that Rad-score (OR: 4.066, 95% CI: 3.495–8.790) and renal vein invasion (OR: 12.914, 95% CI: 1.118–149.112) were independent predictors and used to develop the radiomics model. The radiomics model showed good calibration and discrimination and yielded an AUC of 0.872 (95% CI: 0.821–0.923), 0.865 (95% CI: 0.800–0.930), and 0.848 (95% CI: 0.728–0.967) in three cohorts, respectively. DCA showed the clinical usefulness of the radiomics model in predicting the Leibovich risk groups.

**Conclusions:**

The radiomics model can be used as a non-invasive and useful tool to predict the Leibovich risk groups for localized ccRCC patients.

**Critical relevance statement:**

The triphasic CT-based radiomics model achieved favorable performance in preoperatively predicting the Leibovich risk groups in patients with localized ccRCC. Therefore, it can be used as a non-invasive and effective tool for preoperative risk stratification of patients with localized ccRCC.

**Key points:**

• The triphasic CT-based radiomics signature achieves better performance than the single-phase radiomics signature.

• Radiomics holds prospects in preoperatively predicting the Leibovich risk groups for ccRCC.

• This study provides a non-invasive method to stratify patients with localized ccRCC.

**Graphical Abstract:**

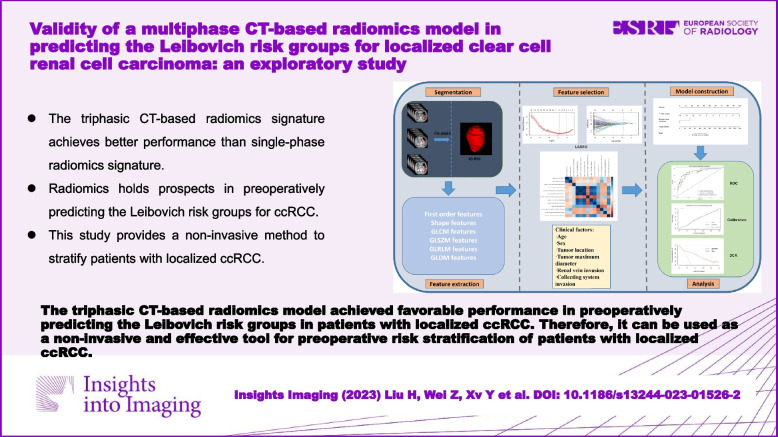

**Supplementary Information:**

The online version contains supplementary material available at 10.1186/s13244-023-01526-2.

## Introduction

Renal cell carcinoma (RCC) is the most common kidney malignancy, accounting for approximately 90% of all kidney malignancies. Clear cell renal cell carcinoma (ccRCC) is the most predominant and malignant subtype of RCC, accounting for approximately 70–80% of RCC [1]. With the development of abdominal imaging technology, a large amount of primary localized ccRCC has been detected [2]. For localized ccRCC, surgery is the main treatment approach in the clinic. However, up to 30% of patients will experience recurrence or metastasis after surgery, especially in patients with advanced localized ccRCC [3, 4]. The Leibovich scoring system based on tumor stage, regional lymph node status, tumor size, histological nuclear grade, and histologic tumor necrosis is one of the most widely utilized prognostic scoring systems that can be used to risk-stratify patients to assist in clinical trials for primary localized ccRCC developed by the Mayo Clinical Center [5, 6]. For primary localized ccRCC, the Leibovich scoring system can be used to predict progression to metastatic disease thereby stratifying patients to adjuvant treatment regimens after surgery [5]. In the era of individualized medicine, it is critical to precisely predict the risk of patients' disease progression before surgery to guide patient management, assist clinicians in clinical decision-making and patient counseling, and formulate risk-appropriate adjuvant and follow-up strategies [3, 5, 7]. However, this information is only available after postoperative pathologic assessment. There is a lack of noninvasive and effective methods to stratify patients with localized ccRCC preoperatively.

Computed tomography (CT) is an essential tool for the evaluation of patients with localized ccRCC in clinical, and at the time of primary diagnosis is inadequate to predict the risk of disease progression after surgery [8]. Radiomics is an emerging non-invasive approach to characterize the heterogeneity and aggressiveness of tumors, which extract a large number of mathematical features by transforming medical images into mineable high-dimensional data [9, 10]. Studies have shown that radiomics are an increasingly promising add-on to non-invasively provide clinically important information in many types of tumors, such as lung cancer [11], liver cancer [12], breast cancer [13], ovarian cancer, and bladder cancer [14, 15]. In addition, radiomics has been reported to be successfully applied in renal tumors, such as benign and malignant differentiation of RCC, subtype classification of RCC, clinical TNM stage prediction of RCC, and pathological Fuhrman nuclear grading or the World Health Organization and International Society of Urological Pathology (WHO/ISUP) grading system prediction of ccRCC [16–20]. However, prognostic studies of radiomics in patients with ccRCC are rarely reported. Currently, there are no studies to predict the Leibovich score risk groups based on CT radiomics for localized ccRCC patients.

In this study, 425 patients with primary localized ccRCC from two medical centers were retrospectively enrolled. The purpose is to develop and validate a multiphase CT-based radiomics model for preoperative risk stratification of patients with localized ccRCC.

## Materials and methods

### Patient

This study was approved by the Ethics Committee of two medical institutions, and the requirement for informed consent was waived due to the retrospective nature of the study.

**Fig. 1:**
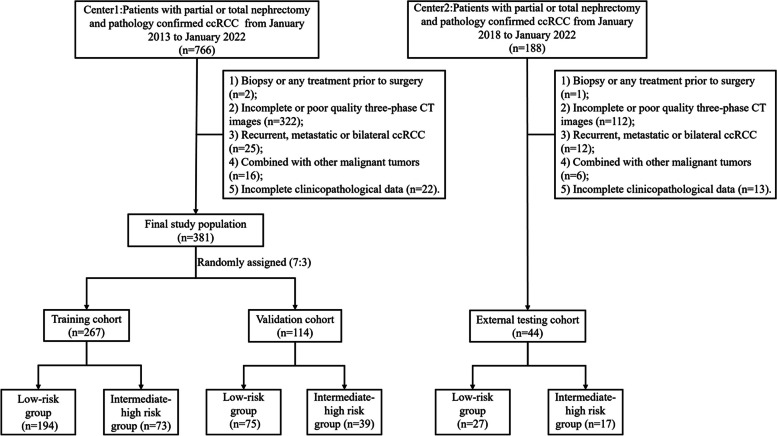
The flowchart of the patient recruitment process

Data for this study were obtained retrospectively from two independent clinical medical centers, which were from the First Hospital of Chongqing Medical University (center 1) from January 2013 to January 2022 and the Second Hospital of Chongqing Medical University (center 2) from January 2018 to January 2022, respectively. According to the following inclusion criteria: (1) Patients who underwent partial or total nephrectomy and postoperative pathology confirmed ccRCC; (2) Patients underwent plain and three-phase enhanced CT scan (unenhanced phase, arterial phase, portal-venous phase, and excretory phase) within 2 weeks before surgery. The exclusion criteria were as follows: (1) Patients who underwent biopsy or any treatment before surgery; (2) Patients with incomplete or poor quality plain and three-phase enhanced CT images; (3) Patients with recurrent, metastatic or bilateral ccRCC; (4) Patients combined with other malignant tumors; (5) Patients without complete clinicopathological data. All pathological diagnosis was rechecked by a pathologist with 10 years of genitourinary experience. Finally, a total of 425 patients were enrolled in this study. The enrolled 381 patients from center 1 were randomized 7:3 into the training cohort and validation cohort, whereas 44 patients from center 2 were used as an independent external testing cohort. The patient recruitment pathway is shown in Fig. 1.

### CT acquisition and evaluation of clinical imaging information

All CT images were acquired by using 64-channel CT scanners (Discovery 750 HD, GE Healthcare, Milwaukee, WI) or 128-channel CT scanners (Siemens Healthcare, Germany) in the axial plane with a tube voltage of 120–140 kV, tube current of 220–300 mAs, matrix of 512 × 512, gantry rotation time of 0.5 s, and section thickness of 5 mm. After intravenous administration of iohexol (300 mg/mL at a rate of 3.0 mL/s, followed by a 30-mL saline flush), contrast-enhanced CT images were captured. The total contrast volume for each kilogram was 1.5 mL. First, unenhanced phase (U) CT of the abdomen (from the superior border of the kidneys to the pubic symphysis) was acquired. Afterward, arterial phase (A), portal-venous phase (V), and excretory phase (E) were acquired at the 25 s, 70 s, and 300 s following the administration of the contrast agent, respectively. Two radiologists (reader1 and reader2) with 10 and 5 years of diagnostic abdominal imaging experience, respectively, evaluated the imaging features: tumor location (left or right), tumor maximum diameter (maximum diameter of the lesion), renal vein invasion (absent or present, tumor thrombosis was seen in the renal vein and inferior vena cava), collecting system invasion (absent or present, tumor infiltrates renal pelvis or renal cone, or the collecting system is distorted by compression), and the discrepancies were re-evaluated through a third senior radiologist (FJL chief of the radiology department).

### Segmentation of tumor and extraction of radiomics features

The 3D volumes of tumors were manually delineated as tumor regions of interest (ROI) by two radiologists using the ITK-SNAP software at three-phase CT images. More information can be found in the supplementary material (Segmentation of tumor). Using spline interference, all images were resampled to symmetrical voxels of 1 × 1 × 1 mm^3^. All radiomics features were extracted from three-phase CT images (unenhanced phase, arterial phase, portal-venous phase) of tumor ROI using the "open-source python package" of pyradiomics in Python, according to the guidelines of the Image Biomarker Standardization Initiative (IBSI) [21, 22]. More details of radiomics feature processing can be found in the supplementary material (The PyRadiomics setting). The extracted radiomics features included the following: first-order features; shape features; gray level cooccurrence matrix (GLCM) features; gray level size zone matrix (GLSZM) features; gray level run length matrix (GLRLM) features and gray level dependence matrix (GLDM) features. All radiomics features were standardized separately using *z*-scores normalization. Finally, 1218 radiomics features were extracted from the tumor ROI of each phase CT image, including 1218 unenhanced phase, 1218 arterial phase, and 1218 portal-vein phase radiomics features. Then a total of 3654 radiomics features were combined together for further analysis from the three-phase CT images (unenhanced phase, arterial phase, portal-venous phase). More information can be found in Supplementary Table 1.

### Clinicopathological data and the Leibovich risk groups

Baseline clinicopathological information included: age, sex, tumor size, pathological regional lymph node status (PN stage), pathological T category of tumor (PT stage), pathological nuclear grade (IUSP grade), and histological tumor necrosis. An updated version of the prognostic scoring system named Leibovich 2018 is presented based on a recent study, but it requires extensive external validation to demonstrate the clinical utility of the model [23]. Therefore, we applied the most common Leibovich 2003 scoring system. According to previous studies, patients with localized ccRCC were categorized into two risk groups: low-risk group (Leibovich < 3 score) and intermediate-high risk group (Leibovich ≥ 3 score) by assessing tumor size, PT stage, PN stage, nuclear grade, and pathological tumor necrosis [3–5]. The Leibovich score criteria are shown in Supplementary Fig. 1.

### Feature selection and construction of radiomics signatures

To select radiomics features that discriminated the low-risk and intermediate-high-risk groups of Leibovich for localized ccRCC patients. First, the ROI of the tumor was outlined by two radiologists (reader1 and reader2) from 30 randomly selected localized ccRCC patients with three-phase CT images to evaluate inter-observer reproducibility. After 2 weeks, the tumor ROI of these 30 ccRCC patients was repeatedly outlined by reder1 to evaluate intra-observer reproducibility. Second, inter- and intra-class correlation coefficients (ICCs) were used to evaluate the inter-observer reliability and intra-observer reproducibility of feature extraction. An ICC greater than 0.75 indicates satisfactory inter- and intra-observer reproducibility. So, radiomics features with good reproducibility for ICC > 0.75 were retained for further analysis. Third, these robust radiomics features were further selected by t-test and the least absolute shrinkage and selection operator (LASSO) regression algorithm with 5-fold cross-validation. Finally, radiomics signatures were constructed using features selected by the LASSO regression algorithm, and the radiomics score (Rad-score) for each patient in the training, validation, and external testing cohorts was calculated by the weight coefficients of features selected by LASSO regression.

### Development of the radiomics model

Multivariate logistic regression of the Rad-score and significant clinical factors (age, sex, tumor location, tumor maximum diameter, renal vein invasion, and collecting system invasion) were used to select independent risk factors for predicting the Leibovich risk groups. A forward-backward stepwise regression based on the minimization Akaike information criterion (AIC) was performed to select the optimal model [24]. Ultimately, the optimal model of incorporating significant clinical factors and Rad-score constructed a radiomics model and visualized as a radiomics nomogram. Furthermore, to facilitate the application of the prediction model, a dynamic nomogram was developed as an interactive application to be published online [25].

### Model performance evaluation and validation

**Fig. 2:**
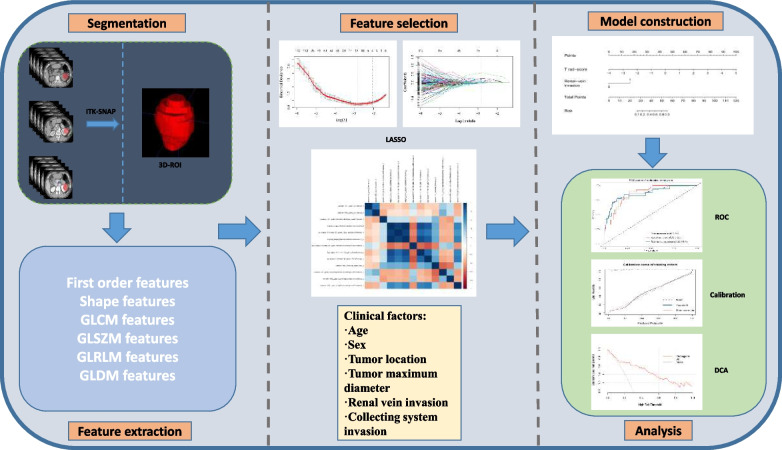
The overall workflow of this study

The discrimination performance of radiomics signatures and the radiomics model was evaluated by the receiver operating characteristic curve (ROC) analysis with the area under the curve (AUC) and 95% confidential interval (95% CI). The cutoff value was identified according to the maximum Youden’s index and used to calculate the sensitivity, specificity, and accuracy in three cohorts. Calibration curves are used to evaluate the consistency between the predicted probability and the actual probability of the radiomics model. Decision analysis curves (DCA) were used to evaluate the clinical usefulness of the radiomics model. The overall workflow of this study is shown in Fig. 2.

### Quality control and transparency

This study strictly adhered to the CLEAR (CheckList for EvaluAtion of Radiomics) reporting guidelines to improve the credibility, reproducibility, and transparency of the study [26]. Detailed information on the CLEAR checklist can be found in the supplemental material (Supplementary Information- CLEAR checklist).

### Statistical analysis

ITK-SNAP (version 3.6.0) was used to outline ROI. Pyradiomics package (version 3.0.1) was used to extract radiomics features. Statistical analysis is performed by R software (version 3.5.1, https://www.r-project.org) and IBM SPSS (version 25; IBM Corporation). A two-sided *p* value less than 0.05 was considered a statistically significant difference. Continuous variables were presented as the means and standard deviation (SD), and categorical variables were presented as frequencies and percentages. Categorical and continuous variables were analyzed using the chi-square test and one-way ANOVA, respectively. The AUC was calculated using the “pROC” package and compared through Delong’s test. The nomogram and calibration curves were plotted using the “rms” package, and the decision curve analysis was performed with the “dca.R” package.

## Results

### Characteristics of patients

In this study, a total of 425 localized ccRCC patients were divided into three cohorts. According to a random ratio of 7:3, the training cohort consisted of 267 ccRCC patients from center 1 and the validation cohort consisted of 114 ccRCC patients from center 1. The external testing cohort consisted of 44 ccRCC patients from center 2. Univariate analysis showed no significant differences in patient age, sex, and tumor location in all three cohorts (*p *> 0.05). There were significant differences in renal vein invasion, collecting system invasion, tumor size, PT stage, IUSP grade, and histological tumor necrosis in all three cohorts (*p *< 0.05). The tumor maximum diameter and PN stage were significantly different in the training and external testing cohort but not in the validation cohort (*p* = 0.536, *p* = 0.080). The demographic characteristics, clinical imaging information, and clinicopathological information of the three cohorts are summarized in Table 1.

**Table 1:** Comparison of clinical and pathological characteristics of three cohorts

**Characteristic**	**Training cohort**	***p* value**	**Validation cohort**	***p* value**	**External testing cohort**	***p* value**
Age	57.7 ± 12.0	0.390	56.8 ± 11.7	0.426	65.0 ± 9.0	0.789
Sex		0.780		0.478		0.283
Male	172 (64.4%)		62 (54.4%)		20 (45.5%)	
Female	95 (35.6%)		52 (45.6%)		24 (54.5%)	
Tumor location		0.939		0.063		0.063
Left	140 (52.4%)		45 (39.5%)		26 (59.1%)	
Right	127 (47.6%)		69 (60.5%)		18 (40.9%)	
Tumor maximum diameter	4.3 ± 2.1	< 0.001	4.6 ± 2.2	0.536	4.9 ± 2.0	0.002
Renal vein invasion		< 0.001		< 0.001		0.003
Absent	256 (95.9%)		105 (92.1%)		39 (88.6%)	
Present	11 (4.1%)		9 (7.9%)		5 (11.4%)	
Collecting system invasion		< 0.001		0.009		0.036
Absent	179 (67%)		63 (55.3%)		29 (65.9%)	
Present	88 (33%)		52 (45.7%)		15 (34.1%)	
Tumor size	3.9 ± 2.0	< 0.001	4.0 ± 1.9	0.002	4.7 ± 1.9	0.015
PT stage		< 0.001		< 0.001		< 0.001
pT1a	168 (62.9%)		63 (55.3%)		17 (38.6%)	
pT1b	48 (18%)		23 (20.2%)		16 (36.4%)	
pT2	9 (3.4%)		5 (4.4%)		6 (13.6%)	
pT3a	39 (14.6%)		19 (16.7%)		5 (11.4%)	
pT3b	3 (1.1%)		4 (3.5%)		0	
pT3c	0		0		0	
pT4	0		0		0	
PN stage		< 0.001		0.080		< 0.001
pN0	256 (95.9%)		110 (96.5%)		37 (84.1%)	
pN1	11 (4.1%)		4 (3.5%)		7 (15.9%)	
pN2	0		0		0	
ISUP grade		< 0.001		< 0.001		< 0.001
I	68 (25.5%)		29 (25.4%)		9 (20.5%)	
II	165 (61.8%)		64 (56.1%)		21 (47.7%)	
III	29 (10.9%)		19 (16.7%)		12 (27.3%)	
IV	5 (1.9%)		2 (1.8%)		2 (4.5%)	
Necrosis		< 0.001		< 0.001		< 0.001
Absent	228 (85.4%)		91 (79.8%)		33 (75%)	
Present	39 (14.6%)		23 (20.2%)		11 (25%)	
Leibovich risk groups						
Low risk	194 (72.7%)		75 (65.8%)		27 (61.4%)	
Intermediate risk	59 (22.1%)		33 (28.9%)		12 (27.3%)	
High risk	14 (5.2%)		6 (5.3%)		5 (11.3%)	

*PT* Pathological T category of the tumor, *PN* Pathological regional lymph node status, *IUSP* The World Health Organization and International Society of Urological Pathology grading system

### Feature selection and radiomics signature construction

**Fig. 3:**
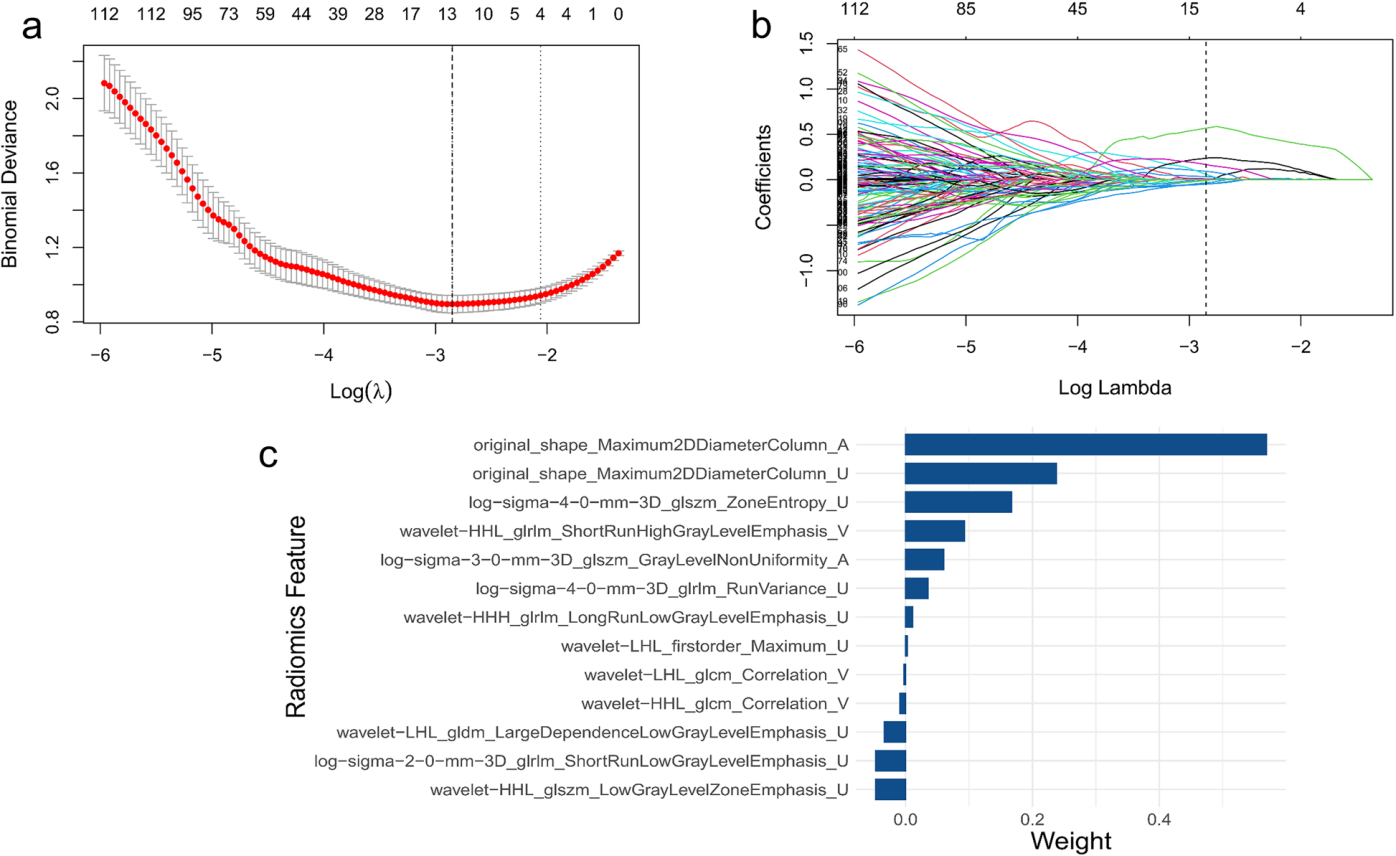
The process of triphasic radiomics features selection and radiomics signature construction by the least absolute shrinkage and selection operator (LASSO) regression algorithm. **a** Based on minimum criteria, we selected tuning parameters (λ) with 5-fold cross-validation. The binomial deviance was plotted versus log(λ). The upper *x*-axis indicates the average number of radiomics features. The lower *x*-axis indicates the log(λ) value. The optimal λ value of 0.0578, with log(λ) = − 2.78 was selected. **b** A coefficient profile plot was generated versus the selected log λ value. **c** The weighting coefficients of each feature. U, unenhanced phase; A, arterial phase; V, portal-venous phase

First, robust features (ICC > 0.75) were selected by inter-observer and intra-observer consistency, including 1009 arterial phase radiomics features, 1037 venous phase radiomics features, 972 unenhanced phase radiomics features, and 3018 triphasic phase radiomics features. Subsequently, 300 arterial phase radiomics features, 324 venous phase radiomics features, 258 unenhanced phase radiomics features and 882 triphasic phase radiomics features were eliminated by *t*-test. Finally, 10 arterial phase radiomics features, 12 venous phase radiomics features, 12 unenhanced phase radiomics features, and 13 triphasic radiomics features were retained as the most valuable radiomics features for predicting the Leibovich risk groups by the LASSO regression algorithm (Fig. 3a, b). The weighting coefficients of 13 triphasic radiomics features are shown in Fig. 3c. The heat map of correlations between 13 triphasic radiomics features is shown in Supplementary Fig. 2. These most worthwhile radiomics features constructed three single-phase radiomics signatures and a triphasic radiomics signature. The ROC curve analysis of four radiomics signatures in the three cohorts is shown in Supplementary Fig. 3a–c. The AUC of the triphasic radiomics signature reached 0.862 (95% CI: 0.809–0.914), 0.853 (95% CI: 0.785–0.921), and 0.837 (95% CI: 0.714–0.959) in the training, validation, and external testing cohorts, respectively, which were higher than the arterial, venous, and unenhanced radiomics signatures (Table 2). Therefore, these triphasic radiomics features were applied to further study. The triphasic radiomics score (T rad-score) was calculated through the weight coefficients of these radiomics features. The formula of the triphasic radiomics score is shown in Supplementary Results. According to Delong’s test, there is only the difference between the triphasic and the arterial phase radiomics signatures (0.853, 0.832, *p* =0.022) was statistically significant in predicting the Leibovich risk groups of patients with localized ccRCC, and no statistically significant difference with the unenhanced phase (0.853, 0.846, *p* = 0.392) and the portal-venous phase radiomics signature (0.853, 0.831, *p* = 0.172) in the validation cohort.

**Table 2:** Results of four radiomics signatures’ predictive ability for predicting the Leibovich risk groups in three cohorts

**Model**	**Cohort**	**AUC (**95% CI**)**	**Sensitivity**	**Specificity**	**Accuracy**	**Cutoff**
U radiomics signature	Training	0.857 (0.803–0.911)	0.726	0.892	0.846	− 0.640
	Validation	0.846 (0.777–0.915)	0.821	0.733	0.763	− 0.910
	External testing	0.801 (0.664–0.939)	0.722	0.769	0.750	− 0.746
A radiomics signature	Training	0.849 (0.795–0.904)	0.726	0.866	0.828	− 0.730
	Validation	0.832 (0.758–0.907)	0.821	0.773	0.789	− 0.870
	External testing	0.803 (0.661–0.946)	0.722	0.846	0.795	− 0.717
V radiomics signature	Training	0.856 (0.804–0.909)	0.753	0.845	0.820	− 0.889
	Validation	0.832 (0.758–0.905)	0.795	0.773	0.781	− 1.004
	External testing	0.823 (0.697–0.948)	0.778	0.769	0.773	− 1.215
T radiomics signature	Training	0.862 (0.809–0.914)	0.753	0.866	0.835	− 0.794
	Validation	0.853 (0.785–0.921)	0.872	0.733	0.781	− 0.999
	External testing	0.837 (0.714–0.959)	0.765	0.852	0.818	− 0.589

*AUC* Area under the receiver operating characteristic curve, *CI* Confidence interval, *U* Unenhanced phase, *A* Arterial phase, *V* Portal-venous phase, *T* triphasic

### The radiomics model development and evaluation

**Fig. 4:**
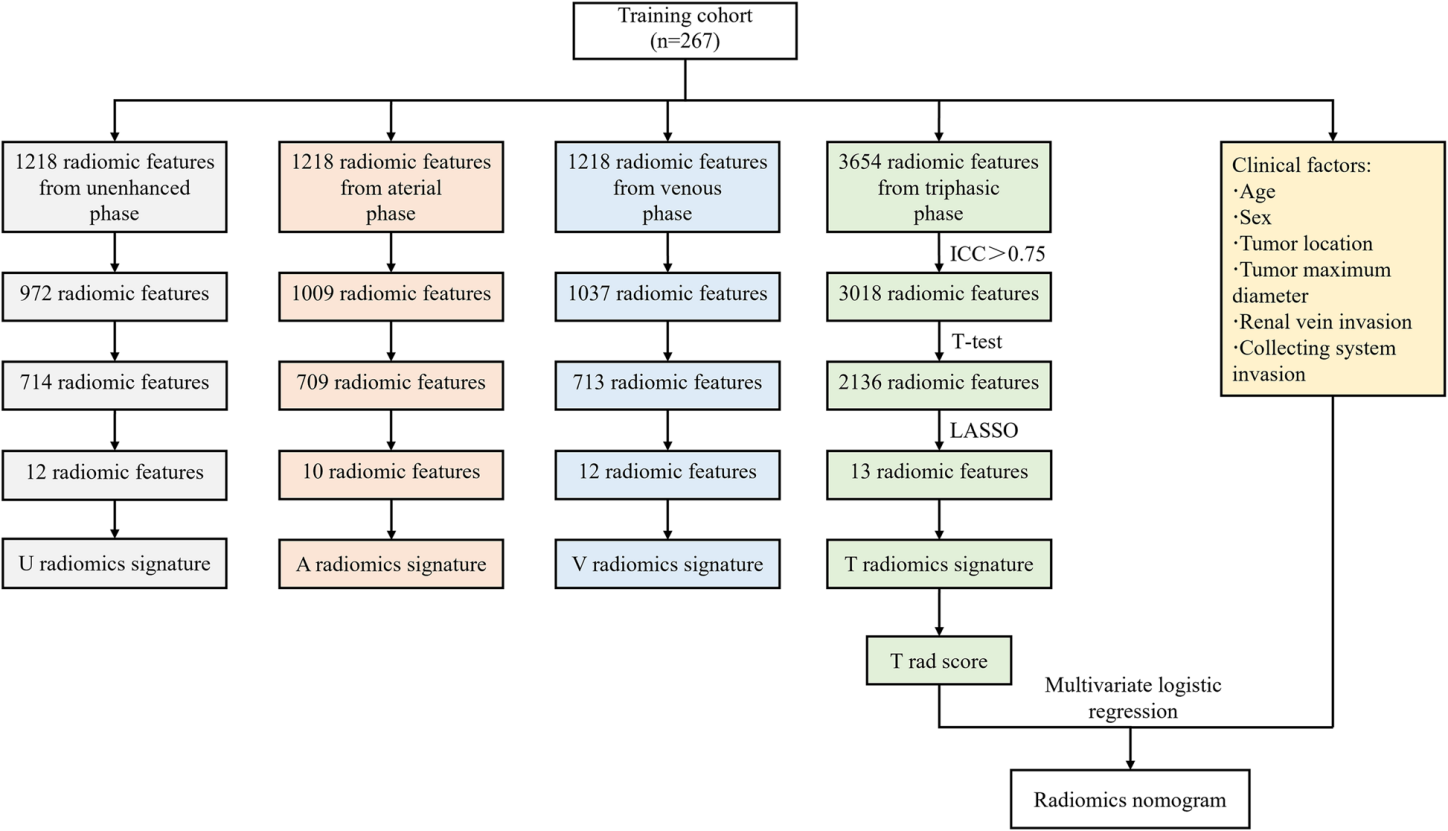
The process of feature selection for constructing four radiomics signatures and the nomogram of the radiomics model construction. U, unenhanced phase; A, arterial phase; V, portal-venous phase; T, triphasic; LASSO, least absolute shrinkage and selection operator, ICC: Intraclass correlation coefficient

**Fig. 5:**
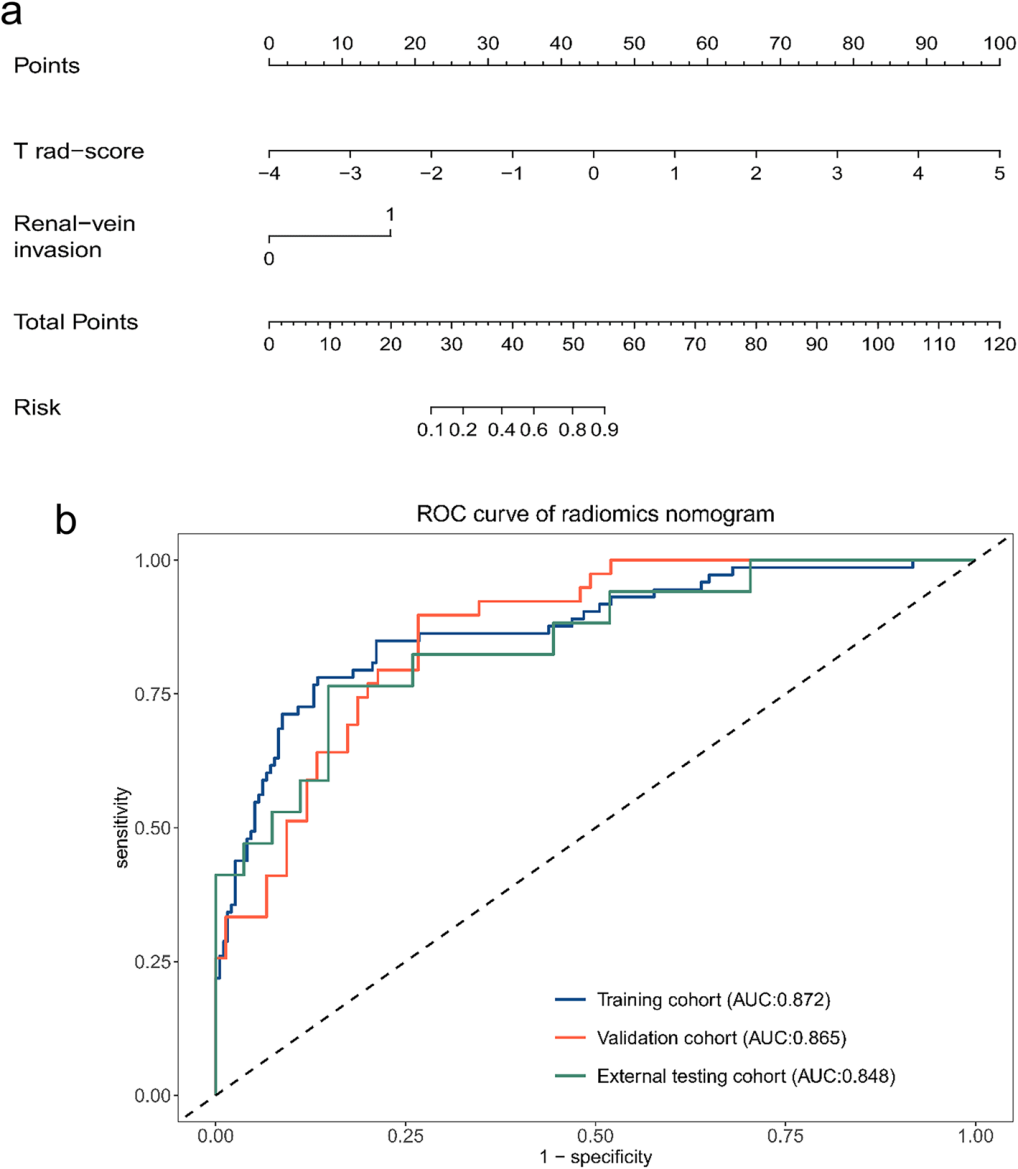
The visualized nomogram of the radiomics model and the receiver operating characteristic (ROC) curve for the radiomics model in three cohorts. **a** The visualized nomogram of the radiomics model, incorporating the triphasic rad-score (T rad-score) and renal vein invasion, developed in the training cohort. **b** The ROC curve of the radiomics model in training, validation, and external testing cohorts, respectively

**Fig. 6:**
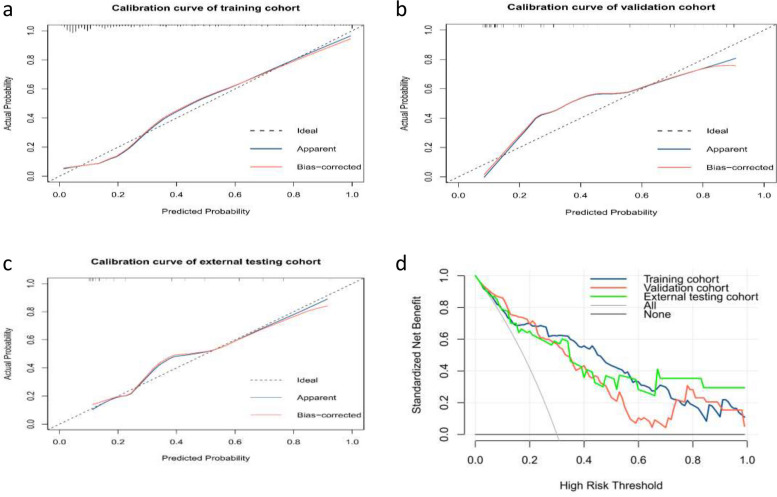
The calibration curves and decision curve analysis of the radiomics model. **a**–**c** The calibration curves of the radiomics model in the training, validation, and external testing cohorts, respectively. **d** DCA for the radiomics model in the training (blue line), validation (red line), and external testing (green line) cohorts, respectively. The *x*-axis shows the threshold probability and the *y*-axis represented the net benefit

Multivariate logistic regression analysis showed that T rad-score (OR: 5.54, 95% CI: 3.495–8.79) and renal vein invasion (OR: 12.91, 95% CI: 1.118–149.112) were independent predictors for predicting the Leibovich risk groups and used to develop a radiomics model and visualized as a radiomics nomogram when AIC takes the lowest value (AIC = 206.1) (Table 3). The process of feature selection and radiomics model construction is shown in Fig. 4. The visualized radiomics nomogram incorporating the T rad-score and renal vein invasion is shown in Fig. 5a. The radiomics model yielded an AUC of 0.872 (95% CI: 0.821–0.923) in the training cohort, 0.865 (95% CI: 0.800–0.930) in validation cohort and 0.848 (95% CI: 0.728–0.967) in the external testing cohort, respectively (Fig. 5b). The predictive performance including AUC, 95% CI, sensitivity, specificity, accuracy, and cutoff of the radiomics model in three cohorts are shown in Table 4. The calibration curve based on the radiomics model showed good calibration in three cohorts (Fig. 6a–c). The DCA showed radiomics model had a higher overall net benefit than the intervention-none strategy or the intervention-all strategy for patients in all three study cohorts across the majority of the range of reasonable threshold probabilities (Fig. 6d).

**Table 3:** Multivariate logistic regression analysis of radiomics score and clinical risk factors in the training cohort

**Characteristics**	**Full multivariate model**			**Reduced multivariate model**		
	**Coefficient**	**OR (95% CI)**	***p* value**	**Coefficient**	**OR (95% CI)**	***p* value**
Intercept	0.802			0.551		
T rad-score	1.962	7.113 (2.517–20.102)	< 0.001	1.712	5.543 (3.495–8.79)	< 0.001
Age	0.006	1.006 (0.976–1.036)	0.707			
Sex	0.185	1.203 (0.563–2.572)	0.633			
Tumor location	0.381	1.463 (0.712–3.007)	0.300			
Tumor maximum diameter	− 0.136	0.873 (0.522–1.458)	0.603			
Renal vein invasion	2.714	15.087 (1.187–191.814)	0.036	2.558	12.914 (1.118–149.112)	0.040
Collecting system invasion	0.102	1.107 (0.498–2.46)	0.802			

*OR* Odds ratio, *CI* Confidence interval, *T rad-score* Triphasic radiomics score

**Table 4:** Results of the radiomics model’s predictive ability for predicting the Leibovich risk groups in three cohorts

**Model**	**Cohort**	**AUC (**95% CI**)**	**Sensitivity**	**Specificity**	**Accuracy**	**Cutoff**
Radiomics model	Training	0.872 (0.821–0.923)	0.781	0.866	0.843	0.308
	Validation	0.865 (0.800–0.930)	0.897	0.733	0.789	0.239
	External testing	0.848 (0.728–0.967)	0.765	0.852	0.818	0.388

*AUC* Area under the receiver operating characteristic curve, *CI* Confidence interval

### Application and publication of the model

To facilitate the application of the prediction model, we have created a dynamic nomogram as an interactive application to visualize our statistical models which is published at https://ccrccleibovichrisk.shinyapps.io/DynNomapps/. The dynamic nomogram is shown in the supplementary material (Supplementary Fig. 4).

## Discussion

In this study, we developed and validated a triphasic CT-based radiomics model which combined significant clinical factors and Rad-score as a non-invasive and novel tool to predict the Leibovich risk groups before surgery for localized ccRCC patients. In addition, our study demonstrated the feasibility and reproducibility of preoperative prediction of Leibovich risk groups in localized ccRCC patients between different medical centers.

Radiomics potentially addresses this problem as an emerging non-invasive tool by extracting a large number of high-throughput radiomics features from the images [27]. In RCC, radiomics has made significant progress in several aspects. Radiomics has been reported to be successfully applied in renal tumors, such as benign and malignant differentiation of RCC [16] and subtype classification of RCC [28, 29]. Therefore, for a renal mass, first, the benign or malignant nature of the mass can be differentiated by radiomics, and if it is a malignant mass, such as the most common RCC, the next step can be to differentiate the subtypes of RCC by radiomics, such as non-clear renal cell carcinoma (non-ccRCC) or clear cell renal cell carcinoma (ccRCC). Therefore, the above steps can be used to diagnose ccRCC preoperatively by radiomics combined with artificial intelligence (AI). Therefore, our present study can further assess tumor aggressiveness to predict the prognosis of patients with ccRCC on this foundation. However, this method still needs to be validated in a large number of prospective and multicenter studies. Previous studies have reported that histological coagulative necrosis, pathological nuclear grading, and TNM stage have been considered significant independent prognostic factors for ccRCC [30, 31]. Kai et al. [32] demonstrated that the CT-based radiomics signature that incorporated radiomics and traditional image features has the potential to be used as a non-invasive tool for preoperative prediction of coagulative necrosis in ccRCC, and obtained the best performance with an AUC of 0.942 in the training set and an AUC of 0.969 in the validation set. In addition, several studies suggest that CT-based texture analysis may prove to be a useful and promising noninvasive tool for assessing ccRCC grading and staging [18–20]. Nevertheless, these are predictive studies of individual prognostic factors, and an integrated system combining multiple independent prognostic variables can achieve higher outcome prediction accuracy and better characterization of tumor heterogeneity and aggressiveness for ccRCC [33]. Ji Whae et al. [34] analyzed 354 patients with ccRCC and constructed an MRI-based radiomics model to prove that preoperative MRI radiomics can accurately predict the stage, size, grade, and necrosis (SSIGN) score of ccRCC. However, this study lacks independent external validation, resulting in insufficient credibility and reproducibility. In addition, CT is cheaper and faster than MRI as the first-line preoperative evaluation tool for patients with ccRCC. Likewise, Yi et al. [35] constructed a radiomics signature consisting of sixteen radiomics features from the nephrographic phase CT images for patients with ccRCC and found CT radiomics signature could be used as a promising non-invasive tool to predict SSIGN risk groups for patients with ccRCC. Our findings are consistent with this study. This study only analyzed those radiomics features extracted from single-phase CT images. However, the radiomics features of our study were extracted from three-phase CT images of patients with ccRCC and can better characterize the heterogeneity and aggressiveness of the tumor. Furthermore, our study was focused on primary localized ccRCC and the endpoint of our study is metastasis-free survival rather than cancer-specific survival, which can better guide clinical decision-making and knowledge of disease progression, as well as the formulation of risk-appropriate follow-up and adjuvant treatment strategies [3–5]. Moreover, according to a recent study, the static nomogram has many limitations, such as not being easily updated; excessive reliance on markers and scores on the axes, which may lead to unstable results; no reporting standards for the nomogram, even if adjusting the graphical ratios at the time of printing may lead to inaccurate scores; and a lack of reproducibility and clinical utility [36]. So, to facilitate the application of the prediction model, we have created a dynamic nomogram as an interactive application to visualize our statistical models which is published at https://ccrccleibovichrisk.shinyapps.io/DynNomapps/. This makes it easier to apply clinically [25]. However, our current study is only a preliminary exploratory study, and a large number of prospective multicenter studies are needed to further validate our model in the future.

In the present study, we extracted 1218 and 3654 radiomics features from monophasic CT and triphasic CT images of each ccRCC patient, respectively, and finally, filtered 13 radiomics features by LASSO regression algorithm to construct radiomics signatures to predict the Leibovich risk groups, including 2 arterial phase features, 3 venous phase features and 8 unenhanced phase features, with AUC of 0.862, 0.853, and 0.837 in the training, validation and external testing cohorts, respectively. We demonstrated that the triphasic radiomics signature achieves higher predictive performance and better characterization of tumor heterogeneity and aggressiveness than single-phase radiomics signatures in predicting Leibovich risk groups for ccRCC patients.

We finally retained radiomics features including shape features, first-order features, and higher-order texture features, and the highest proportion of them was the original_shape_Maximum2DDiameterColumn feature, consistent with previous studies that larger tumors have more aggressive and worse prognostic performance for ccRCC patients [37, 38].

According to previous studies, prognostic factors for patients with RCC include anatomical, histological, clinical, and molecular factors of the tumor [39]. Renal vein invasion is one of the significant clinical prognostic factors of RCC and is associated with aggressiveness and poor prognosis of RCC. The venous invasion has been reported in approximately 4–10% of RCC cases [40, 41]. Venous invasion is a poor prognostic sign and can increase the risk of tumor metastasis [42, 43]. Our study showed that the ultimate radiomics model incorporating renal vein invasion and Rad-score yielded the highest predictive accuracy in predicting the Leibovich risk groups with AUCs of 0.872, 0.865, and 0.848 in the three cohorts, respectively. Meanwhile, the triphasic radiomics signature performed similarly to the radiomics model in predicting the Leibovich risk groups with no statistically significant differences according to Delong’s test.

DCA is a statistical technique for the evaluation of tests or models that focuses on decisions and outcomes. A decision curve is used to evaluate whether a model or test would be of benefit in the clinic. If results are positive, then the model or test can be used with appropriate patients as part of shared decision-making in the clinic [44, 45]. In our study, the DCA showed radiomics model had a higher overall net benefit than the intervention-none strategy or the intervention-all strategy for patients in all three study cohorts across the majority of the range of reasonable threshold probabilities.

A recent updated version of the Leibovich scoring model, namely the Leibovich 2018, identified routinely available clinical and pathologic features that can accurately predict progression and death from RCC following surgery [23]. However, the Leibovich 2003 version is the most commonly used prognostic scoring system in clinical practice for localized ccRCC. In addition, the Leibovich 2018 model requires a large amount of external validation to demonstrate the model's clinical utility. In combination with the above reasons, we studied only the Leibovich 2003 prognostic scoring system.

Based on previous studies, the Leibovich prognostic scoring system divided patients with localized ccRCC into three risk groups: low-risk, intermediate-risk, and high-risk groups with five-year metastasis-free probabilities of 97.1% (low-risk), 73.8% (intermediate-risk), and 31.2% (high-risk), respectively [5]. In this study, we combined the Leibovich intermediate-risk group and the high-risk group together for study. The main reasons for combining the intermediate-high-risk group in this study are: first, due to the limitation of sample size, the sample size of patients in the low-risk group is large, while the sample size of patients in the high-risk group is small, and if this sample is further divided into intermediate-risk and high-risk groups for the study may result in too small a sample size, leading to overfitting and affecting the reliability and stability of the statistical analysis. Therefore, the intermediate-high-risk group was combined in order to increase the number of training samples and improve the robustness and stability of the prediction model. Second, in the clinical setting, patients of the Leibovich low-risk group are at a lower risk of postoperative metastasis, while patients of the Leibovich intermediate-high-risk groups are more likely to experience metastasis. For patients of the intermediate-high-risk group, more aggressive treatment options can be selected, such as the scope and manner of surgical resection needs to be more aggressive to reduce the risk of postoperative recurrence and metastasis. In contrast, for low-risk patients, unnecessary examinations and treatments can be reduced, so as to optimize the use of resources. Therefore, it is significant to accurately distinguish the Leibovich low-risk group from the intermediate-high-risk group for better-individualized treatment.

Based on some recent systematic reviews, our study has many strengths in methodology that make our predictive models have better generalizability, reproducibility, and clinical utility [46–48]. As detailed in the supplementary material (Supplementary Table 2).

This study has several limitations. Firstly, the sample size of this two-center retrospective study was limited, which may lead to selection bias. Therefore, a prospective study with a large sample is needed in the future. Secondly, the sample in this study was not uniformly distributed, and the sample of the high-risk group was small approximately 6.56% of the total sample size. Therefore, this study only explored the prediction of the low-risk and intermediate-high-risk groups of Leibovich, and the prediction of the intermediate-risk and high-risk groups of Leibovich is needed in the future. So, there is a need to continue to expand the sample size in the future, especially for patients in the Leibovich high-risk group. Thirdly, the ROI was manually segmented by radiologists, which is time-consuming and easily leads to subjective bias. As outlined and recommended in the very recent ESR and EORTC consensus paper on standardized lesion segmentation for imaging biomarker quantitation, the manual segmentation should be checked and corrected by a second observer to reach a consensus on the delineations [49]. Therefore, our study did not use a consensus-based segmentation method and was independently segmented by radiologists. Because according to a recent study showing that consensus-based segmentation has significant reproducibility issues in radiomics [50]. Future research should focus on developing automated segmentation methods to improve reliability and reproducibility. Finally, multi-omics research is the future trend and more biomarkers should be incorporated to improve the performance of prediction, such as genomics and pathomics [51, 52].

In conclusion, we developed and validated a triphasic CT-based radiomics model incorporating radiomics features and significant clinical factors, and achieved a favorable performance in preoperatively predicting the Leibovich low-risk and intermediate-high-risk groups in patients with localized ccRCC. Meanwhile, we demonstrated that a multiphase CT-based radiomics method can be used for preoperative risk stratification of patients with localized ccRCC. Our study conforms to standard radiomics processes, addresses some common shortcomings, and achieves a higher radiomics quality score. Besides, this visualized nomogram of the radiomics model can be used as a non-invasive and effective tool to facilitate clinical decision-making and monitoring of disease progression.

### Supplementary Information

**Additional file 1:** **Supplementary Fig. 1.** The Leibovich score criteria. **Supplementary Table 1.** Radiomics features extracted from single-phase CT images and triphasic CT images. **Supplementary Fig. 2.** Heat map of correlation between triphasic radiomics features selected by the least absolute shrinkage and selection operator (LASSO) regression algorithm. **Supplementary Fig. 3.** The ROC curve analysis of four radiomics signatures in the three cohorts.**Supplementary Fig. 4.** Dynamic nomogram (online version) for patients with localized ccRCC that predicts the Leibovich risk groups. **Supplementary Results.** The formula of the triphasic radiomics score. **Supplementary Table 1.** Radiomics features extracted from single-phase CT images and triphasic CT images. **Supplementary Table 2.** This studies' methodological strengths. **Other Supplements1.** Segmentation of tumor. **Other Supplements2.** The PyRadiomics setting. **Supplementary Fig. 5.** Manual tumor segmentation was conducted on axial slices of renal lesions.

## Data Availability

The datasets analyzed during the current study are not publicly available due to local restrictions of data protection but are available from the corresponding author upon reasonable request.

## Abbreviations

AIC: Akaike information criterion
AUC: Area under the curve
CI: Confidence interval
CLEAR: CheckList for EvaluAtion of Radiomics
CT: Computed tomography
DCA: Decision curve analysis
GLCM: Gray-level cooccurrence matrix
GLDM: Gray-level dependence matrix
GLRLM: Gray-level run-length matrix
GLSZM: Gray-level size-zone matrix
ICC: Intraclass correlation coefficient
LASSO: Least absolute shrinkage and selection operator
OR: Odds ratio
Rad-score: Radiomics score
RCC: Renal cell carcinoma
ROC: Receiver operating characteristic
ROI: Region of interest
