# Supplementary material for: Validity of a multiphase CT-based radiomics model in predicting the Leibovich risk groups for localized clear cell renal cell carcinoma: an exploratory study
Source: Insights Imaging. 2023 Oct 10;14:167. doi: 10.1186/s13244-023-01526-2 (PMC10564697; doi:10.1186/s13244-023-01526-2)
Supplement: Supplementary file 1 — Additional file 1: Supplementary Fig. 1. The Leibovich score criteria. Supplementary Table 1. Radiomics features extracted from single-phase CT images and triphasic CT images. Supplementary Fig. 2. Heat map of correlation between triphasic radiomics features selected by the least absolute shrinkage and selection operator (LASSO) regression algorithm. Supplementary Fig. 3. The ROC curve analysis of four radiomics signatures in the three cohorts.Supplementary Fig. 4. Dynamic nomogram (online version) for patients with localized ccRCC that predicts the Leibovich risk groups. Supplementary Results. The formula of the triphasic radiomics score. Supplementary Table 1. Radiomics features extracted from single-phase CT images and triphasic CT images. Supplementary Table 2. This studies' methodological strengths. Other Supplements1. Segmentation of tumor. Other Supplements2. The PyRadiomics setting. Supplementary Fig. 5. Manual tumor segmentation was conducted on axial slices of renal lesions. [file 13244_2023_1526_MOESM1_ESM.docx]

**Validity of a multiphase CT-based radiomics model in predicting the Leibovich risk groups for localized clear cell renal cell carcinoma: an exploratory study**

**ELECTRONIC SUPPLEMENTARY MATERIAL**

**Supplementary Fig. 1:** The Leibovich score criteria.

**Supplementary Table 1:** Radiomics features extracted from single-phase CT images and triphasic CT images.

**Supplementary Fig. 2:** Heat map of correlation between triphasic radiomics features selected by the least absolute shrinkage and selection operator (LASSO) regression algorithm.

**Supplementary Fig. 3:** The ROC curve analysis of four radiomics signatures in the three cohorts.

**Supplementary Fig. 4:** Dynamic nomogram (online version) for patients with localized ccRCC that predicts the Leibovich risk groups.

**Supplementary Results:** The formula of the triphasic radiomics score.

**Supplementary Table 2:** This studies' methodological strengths.

**Other Supplements1:** Segmentation of tumor.

**Other Supplements2:** The PyRadiomics setting**.**

**Supplementary Fig. 5:** Manual tumor segmentation was conducted on axial slices of renal lesions.


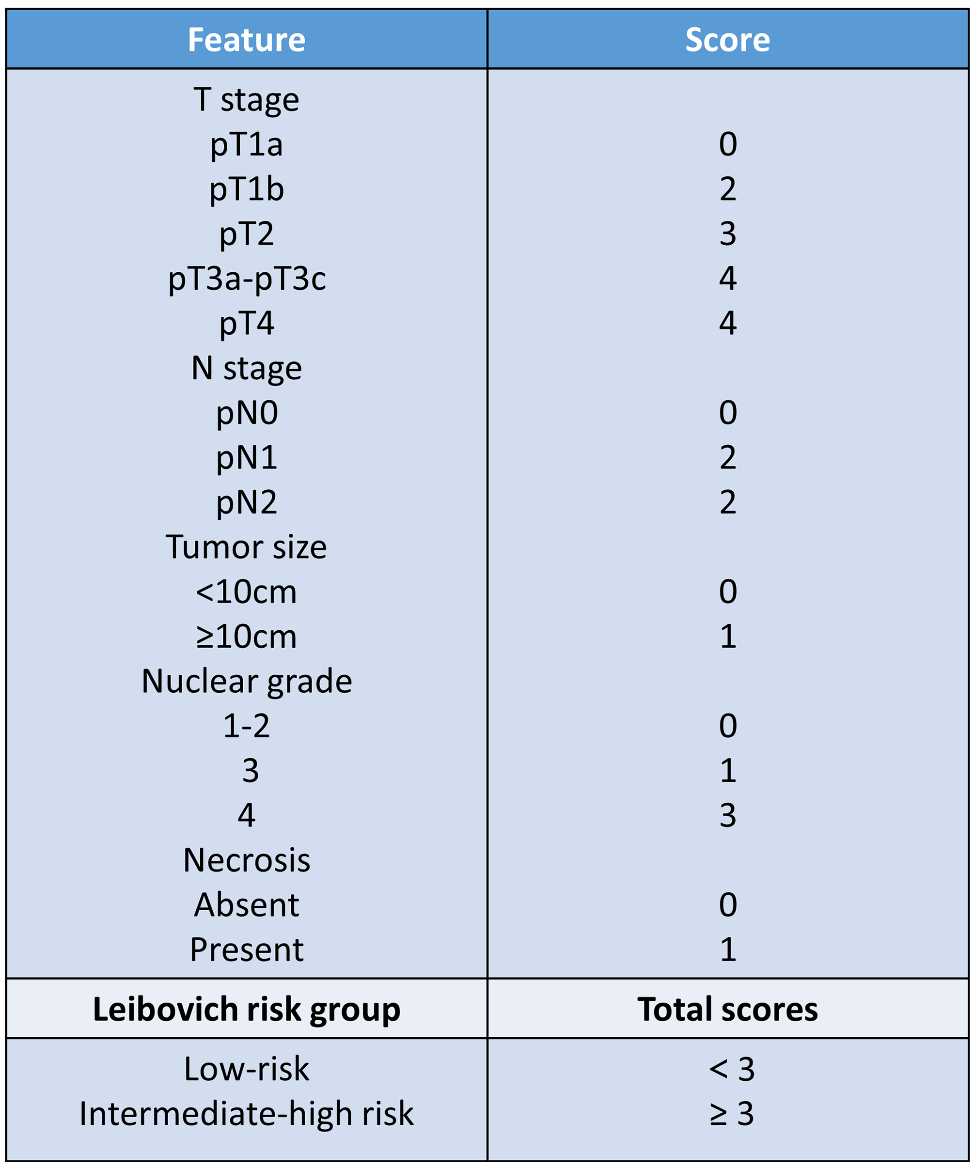


Supplementary Fig. 1: The Leibovich score criteria.

Supplementary Table 1: Radiomics features extracted from single-phase CT images and triphasic CT images

| **Single phase (1218)** | **Triphasic (3654)** |
| --- | --- |
| 1. Shape features: 14; 2. First-order features: 18; 3. Gray Level Cooccurrence Matrix (GLCM): 22; 4. Gray Level Run Length Matrix (GLRLM): 16; 5. Gray Level Size Zone Matrix (GLSZM): 16; 6. Gray Level Dependence Matrix (GLDM): 14; 7. Wavelet features: 688; 8. Laplacian of Gaussian-filtered features: 430. | 1. Shape features: 42; 2. First-order features: 54; 3. Gray Level Cooccurrence Matrix (GLCM): 66; 4. Gray Level Run Length Matrix (GLRLM): 48; 5. Gray Level Size Zone Matrix (GLSZM): 48; 6. Gray Level Dependence Matrix (GLDM): 42; 7. Wavelet features: 2064; 8. Laplacian of Gaussian-filtered features: 1290. |

All radiomics features were extracted from the original and filtered images (5 Laplace of Gaussian filter and 8 Wavelet transform) for each tumor. Patient's single-phase CT image (unenhanced phase, arterial phase, and portal-venous phase).


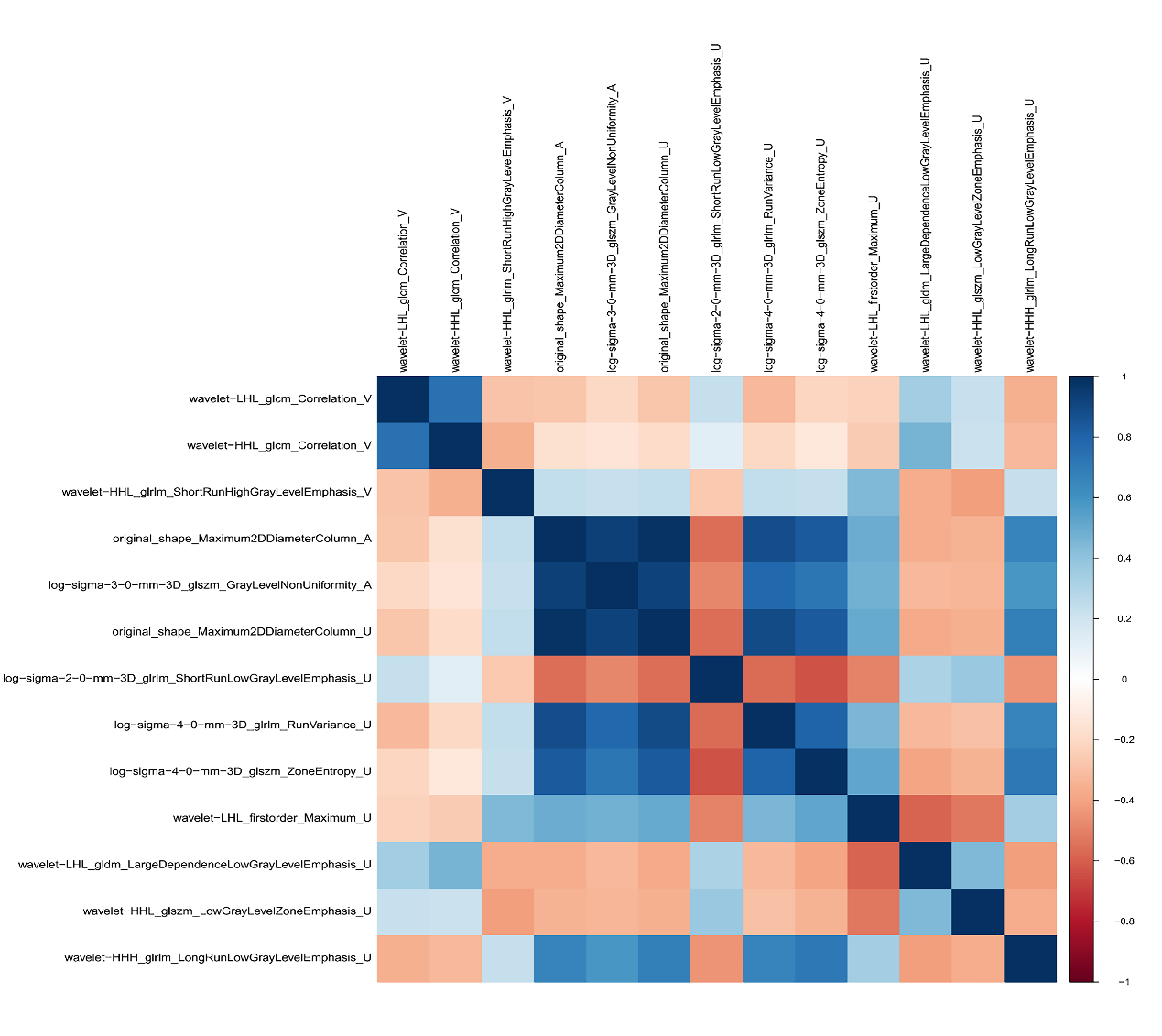


Supplementary Fig. 2: Heat map of correlation between triphasic radiomics features selected by the least absolute shrinkage and selection operator (LASSO) regression algorithm. U: unenhanced phase, A: arterial phase, V: portal-venous phase.


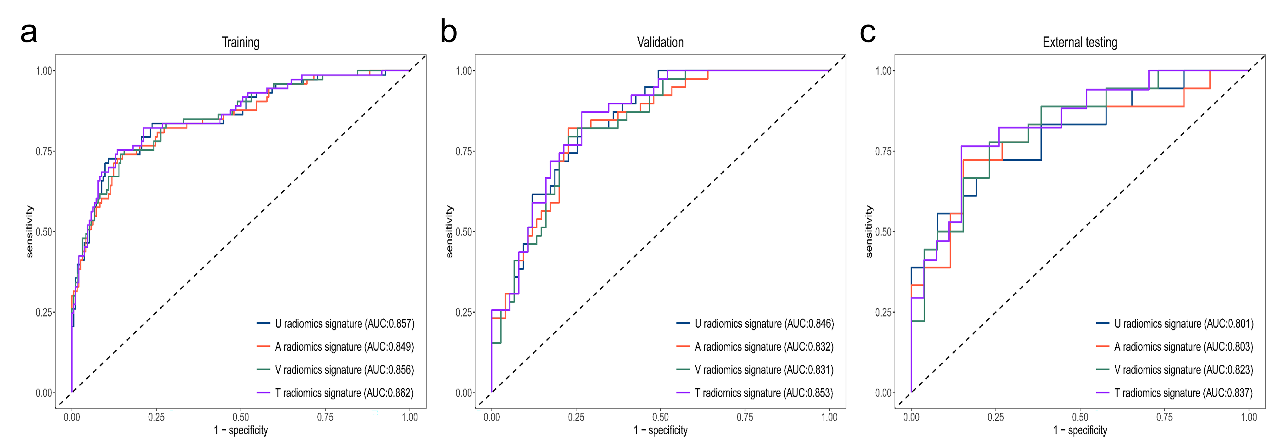


Supplementary Fig. 3: The ROC curve analysis of four radiomics signatures in the training (a), validation (b), and external testing (c) cohort, U: unenhanced phase, A: arterial phase, V: portal-venous phase, T: triphasic.


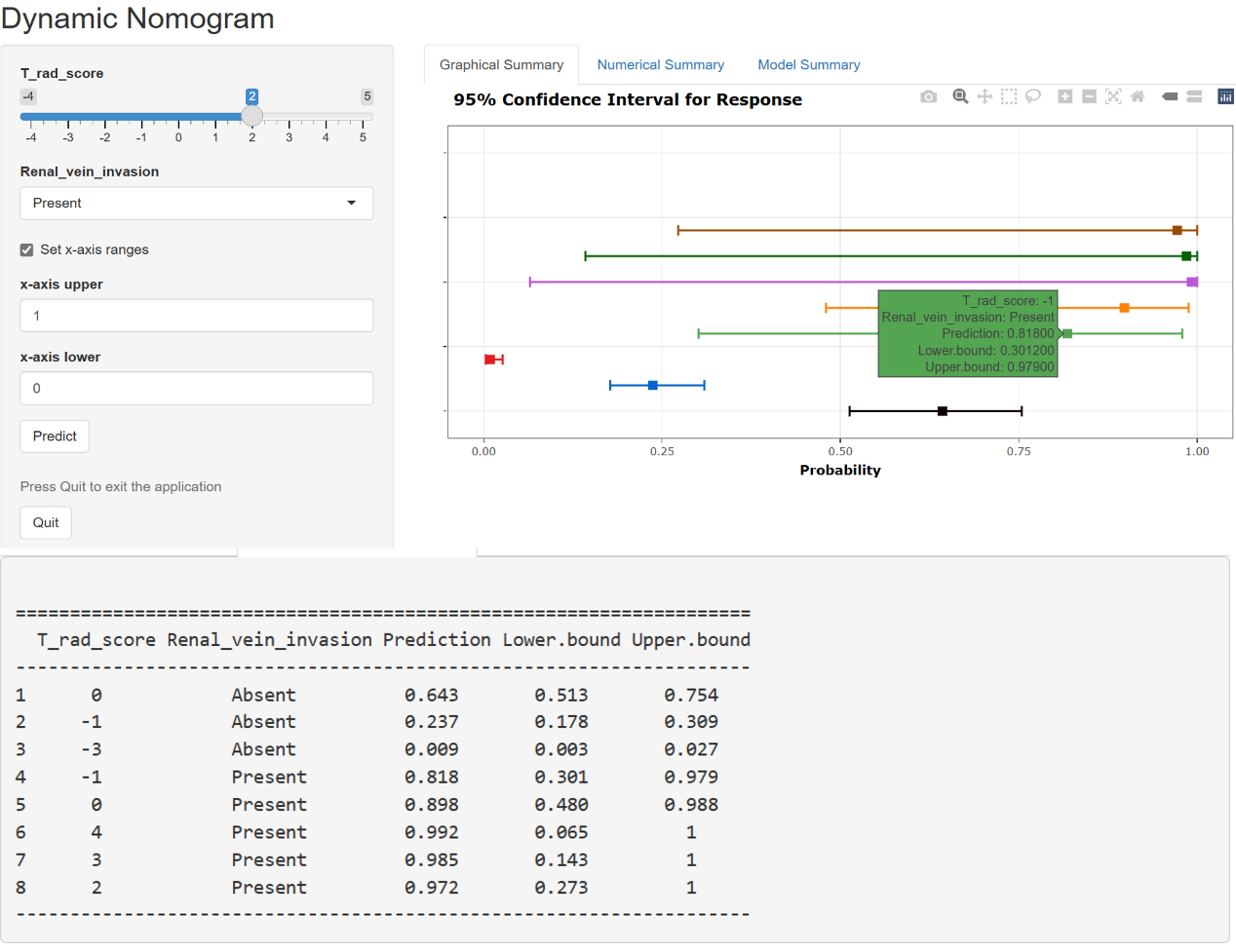


Supplementary Fig. 4: Dynamic nomogram (online version) for patients with localized ccRCC that predicts the Leibovich risk groups.

The Rad-score formula is as shown below:

Rad-score = -1.1457 +

-0.0469*wavelet-HHL_glszm_LowGrayLevelZoneEmphasis_U +

-0.0469*log-sigma-2-0-mm-3D_glrlm_ShortRunLowGrayLevelEmphasis_U +

-0.0334*wavelet-LHL_gldm_LargeDependenceLowGrayLevelEmphasis_U +

-0.0086*wavelet-HHL_glcm_Correlation_V +

-0.0026*wavelet-LHL_glcm_Correlation_V +

0.0023*wavelet-LHL_firstorder_Maximum_U +

0.0107*wavelet-HHH_glrlm_LongRunLowGrayLevelEmphasis_U +

0.0352*log-sigma-4-0-mm-3D_glrlm_RunVariance_U +

0.0598*log-sigma-3-0-mm-3D_glszm_GrayLevelNonUniformity_A +

0.0925*wavelet-HHL_glrlm_ShortRunHighGrayLevelEmphasis_V +

0.167*log-sigma-4-0-mm-3D_glszm_ZoneEntropy_U +

0.2375*original_shape_Maximum2DDiameterColumn_U +

0.5685*original_shape_Maximum2DDiameterColumn_A

Supplementary Results: The formula of the triphasic radiomics score, U: unenhanced phase, A: arterial phase, V: portal-venous phase.

Supplementary Table 2: This studies' methodological strengths

| **Strengths** | **Descriptions** |
| --- | --- |
| Strength 1 | Large sample size, a total of 425 ccRCC patients from two independent medical centers |
| Strength 2 | A two-center study, which increased the reliability of the findings |
| Strength 3 | Using multi-phase CT images and integrating significant clinical data to develop a combined model, which helped to improve the accuracy and clinical utility of the predictive model |
| Strength 4 | Using internal and independent external validation to evaluate the performance of the predictive models, which increases the credibility and generalizability of the findings |
| Strength 5 | Integrating radiomics features and clinical data, and this integrated analysis approach is expected to improve the integrative ability of predictive models to better guide clinical practice |
| Strength 6 | Following a standardized methodology in terms of image analysis, which helped to improve the reliability and reproducibility of the study |
| Strength 7 | Having created a dynamic nomograms as interactive applications to visualise statistical models and is published online, this makes it easier to apply clinically and increased clinical utility |
| Strength 8 | Sharing code and models so that to contribute to transparency and scientific sharing, and other researchers can better understand our methods and implementations, and conduct further research |

**Other Supplements1:** Segmentation of tumor.

An independent radiologist (Radiologist 1；Reader 1; with 10-year experience of abdominal imaging diagnosis) manually segmented the tumor region of interest (ROI) using the ITK-SNAP software (version 3.6.0), who was blinded to the pathological results. When delineating the tumor ROI, radiologists first delineate from the arterial phase, followed by delineating the portal-venous phase, and finally the plain phase (unenhanced phase) is outlined layer by layer with reference to the arterial and portal-venous phases. Meanwhile, the integrated coronal/sagittal images provide a more comprehensive understanding of the size, location, and morphology of the tumor, and are measured and judged from different angles and directions, resulting in more accurate tumor cranio-caudal dimension. The final combination of the three dimensions of reconstruction forms the 3D-ROI of the tumor (Supplementary Fig. 5). To evaluate the reproducibility radiomics features and calculate the Inter- and intra- class correlation coefficients (ICCs), 30 localized ccRCC patients were randomly selected for ROIs’ re-segmentation by Reader 1 and another radiologist (Radiologist 2；Reader 2; with 5-year experience of abdominal imaging diagnosis). First, the ROI of the tumor was outlined by two radiologists (reader1 and reader2) from 30 randomly selected localized ccRCC patients to evaluate inter-observer reproducibility. After 2 weeks, the tumor ROI of these 30 ccRCC patients was repeatedly outlined by reder1 to evaluate intra-observer reproducibility. An ICC greater than 0.75 indicates satisfactory inter- and intra-observer reproducibility. So, radiomics features with good reproducibility for ICC > 0.75 were retained for further analysis.


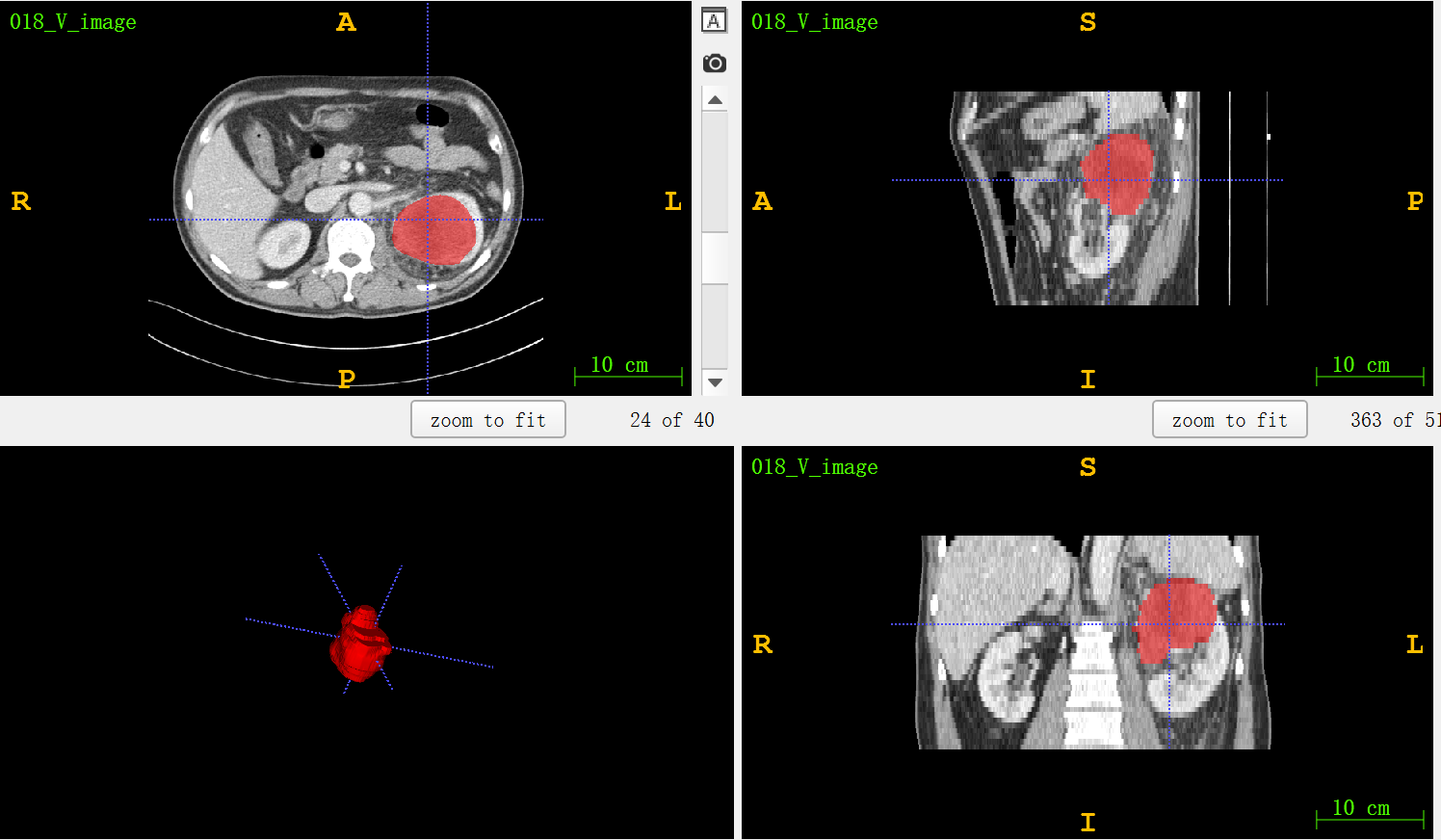


Supplementary Fig. 5: Manual tumor segmentation was conducted on axial slices of renal lesions.

**Other Supplements2:** The PyRadiomics setting**.**

The PyRadiomics setting was shown as follows:

Settings = {'binWidth': 25,

'resampledPixelSpacing': [1, 1, 1],

'interpolator': 'sitkBSpline',

'padDistance': 10,

'sigma': [1.0, 2.0, 3.0, 4.0, 5.0],

'voxelArrayShift': 1000}
